# Devices and furniture for small and sick newborn care: systematic development of a planning and costing tool

**DOI:** 10.1186/s12887-023-04363-w

**Published:** 2023-11-15

**Authors:** Alice Tarus, Georgina Msemo, Rosemary Kamuyu, Donat Shamba, Rebecca P. Kirby, Kara M. Palamountain, Edith Gicheha, Meghan Bruce Kumar, Timothy Powell-Jackson, Christine Bohne, Sarah Murless-Collins, Sara Liaghati-Mobarhan, Alison Morgan, Z. Maria Oden, Rebecca Richards-Kortum, Joy E. Lawn

**Affiliations:** 1 Maternal, Adolescent, Reproductive & Child Health (MARCH) Centre, London School of Hygiene & Tropical Medicine, London, UK; 2 Global Financing Facility, the World Bank Group, Washington, DC USA; 3 Department of Health Systems, Impact Evaluation and Policy, Ifakara Health Institute, Dar Es Salaam, Tanzania; 4 Kellogg School of Management, Northwestern University, Evanston, IL USA; 5 Rice360 Institute for Global Health Technologies, Houston, TX USA; 6 Kenya Medical Research Institute- Wellcome Trust Research Program, Nairobi, Kenya

**Keywords:** Newborn, Neonatal, Costing, Scale-up, Medical Equipment, Medical Devices, Planning, Budgeting, Low- and Middle-Income Countries, Small and Sick Newborn Care

## Abstract

**Background:**

High-quality neonatal care requires sufficient functional medical devices, furniture, fixtures, and use by trained healthcare workers, however there is lack of publicly available tools for quantification and costing. This paper describes development and use of a planning and costing tool regarding furniture, fixtures and devices to support scale-up of WHO level-2 neonatal care, for national and global newborn survival targets.

**Methods:**

We followed a systematic process. First, we reviewed planning and costing tools of relevance. Second, we co-designed a new tool to estimate furniture and device set-up costs for a default 40-bed level-2 neonatal unit, incorporating input from multi-disciplinary experts and newborn care guidelines. Furniture and device lists were based off WHO guidelines/norms, UNICEF and national manuals/guides. Due to lack of evidence-based quantification, ratios were based on operational manuals, multi-country facility assessment data, and expert opinion. Default unit costs were from government procurement agency costs in Kenya, Nigeria, and Tanzania. Third, we refined the tool by national use in Tanzania.

**Results:**

The tool adapts activity-based costing (ABC) to estimate quantities and costs to equip a level-2 neonatal unit based on three components: (1) furniture/fixtures (18 default but editable items); (2) neonatal medical devices (16 product categories with minimum specifications for use in low-resource settings); (3) user training at device installation. The tool was used in Tanzania to generate procurement lists and cost estimates for level-2 scale-up in 171 hospitals (146 District and 25 Regional Referral). Total incremental cost of all new furniture and equipment acquisition, installation, and user training were US$93,000 per District hospital (level-2 care) and US$346,000 per Regional Referral hospital. Estimated cost per capita for whole-country district coverage was US$0.23, representing 0.57% increase in government health expenditure per capita and additional 0.35% for all Regional Referral hospitals.

**Conclusion:**

Given 2.3 million neonatal deaths and potential impact of level-2 newborn care, rational and efficient planning of devices linked to systems change is foundational. In future iterations, we aim to include consumables, spare parts, and maintenance cost options. More rigorous implementation research data are crucial to formulating evidence-based ratios for devices numbers per baby. Use of this tool could help overcome gaps in devices numbers, advance efficiency and quality of neonatal care.

**Supplementary Information:**

The online version contains supplementary material available at 10.1186/s12887-023-04363-w.

## Key findings

**Table:** 

**WHAT WAS KNOWN?**
• Scale-up of WHO level-2 care for small and sick newborns, which includes the provision of continuous positive airway pressure, is receiving increasing global and national attention. This effort has potential to save 747,000 lives annually, in line with Every Newborn Action Plan's fourth target. The target aims to have at least one unit providing level-2 care in 80% of sub-national districts by 2025 • Neonatal devices are lacking or may be donated or procured from high-income settings and hence often unfit for use in low-and middle-income countries (LMIC) due to missing parts and limited training • Health managers/planners are requesting evidence-based approaches to inform equipping newborn care units with appropriate types and quantities of devices and ward furniture • We aimed to co-design, with multi-disciplinary input, a costing tool for newborn care devices and furniture for planners to use at national or subnational levels
**WHAT WAS DONE THAT IS NEW?**
A systematic process was applied: • Review of relevant costing tools, including the UNICEF Oxygen System Planning Tool being the only one that was closely related to our tool’s remit, and we adopted its stepwise approach • Co-design with multi-disciplinary experts to develop a tool for estimating required quantities and incremental costs of ward furniture and devices • Operationalise this tool to estimate set-up costs in Tanzania for 146 Districts and 25 Regional Referral hospitals
**WHAT WAS FOUND?**
• A customisable tool based on activity-based costing approach was developed allowing quantification and costing for a default 40-bed neonatal unit (or multiples thereof), including a ten-bed level-2 (high-dependency) unit for small and sick newborn care. The tool includes three components: (1) ward furniture and fixtures (18 items as default with ratios and unit costs), (2) neonatal devices (16 items as default with ratios and unit costs), (3) device installation training cost estimates for a one-day, onsite face-to-face training • Hence automatically generating cost and procurement estimates are automatically generated into a result dashboard. In Tanzania, it was feasible to collect cost data and generate reports for inclusion in the annual budgeting cycles in less than a week by a non-economic expert user
**WHAT NEXT?**
• The tool provides policymakers with evidence-based cost information to guide budget allocation and catalyse resource mobilisation for scale-up of level-2 care for small and sick newborns • Future iterations of the tool aim to include quantities and costing for consumables, spare parts, and maintenance • There is a need for more robust data on device-to-baby ratios, case mix and more data on unit costs from regions beyond Africa to improve transferability

## Background

Hospital-based care for small and sick newborns has potential to avert approximately 750 000 neonatal deaths each year [1]. Despite the Sustainable Development Goals (SDGs) target for each country to reduce neonatal mortality to < 12 deaths per 1,000 live births by 2030, 63 countries remain off track [2]. To accelerate progress, the *Every Newborn* Action Plan (ENAP) calls for countries to establish at least one unit providing level-2 care plus respiratory support with continuous positive airway pressure (CPAP) (referred to as level-2 care in this paper) in 80% of sub-national units (e.g., districts) by 2025 [3]. Standard care at this level includes thermal care, kangaroo mother care (KMC), assisted feeding and intravenous fluids, safe administration of oxygen, neonatal sepsis management with injected antibiotics, management of neonatal jaundice with phototherapy, management of neonatal encephalopathy, detection of congenital abnormalities and referral or management of birth defects [1, 3]. In addition, the management of respiratory distress with CPAP during transition. Level-2 care alone is not sufficient; linking neonatal care from level-1 to level-3 is necessary to achieve a global neonatal mortality target [1]. Level-1 provides immediate and essential newborn care while level-3 provides intensive and advanced care for very sick babies (Table 1) [1, 4]. Achieving high coverage and high-quality requires the right physical space, the right people with the right training, and the right devices [1, 5, 6].

**Table 1:** World Health Organization definition of levels of newborn care and interventions [4]

Level of newborn care	Scope of care
Level 1: Essential newborn care	Services include immediate care at birth; thorough drying, skin-to-skin contact, delayed cord clamping; resuscitation when needed; early initiation and support for exclusive breastfeeding; routine care (Vitamin K, eye care, vaccinations, weighing, clinical examinations); prevention of mother-to-child transmission of HIV; assessment, management and referral of bacterial infections, jaundice and diarrhoea, feeding problems, birth defects and other problems; pre-discharge advice on mother and baby care and follow-up
Level 2: Special inpatient newborn care	Services include: thermal care; comfort and pain management; kangaroo mother care (< 2500 g irrespective of stability); assisted feeding; safe administration of oxygen; prevention of apnoea; detection and management of neonatal infection, hypoglycaemia, jaundice, anaemia and neonatal encephalopathy; seizure management; safe administration of intravenous fluids; detection and referral management of birth defects.;
+ Transition to intensive care	Continuous positive airway pressure; exchange transfusion; detection and management of necrotizing enterocolitis; specialized follow-up of infants at high risk (including preterm infants)
Level 3: Intensive critical newborn care	Services include: advanced feeding support; mechanical/assisted ventilation, including intubation; screening and treatment for retinopathy of prematurity; surfactant treatment; investigation and management of birth defects; paediatric surgery; genetic services

Abbreviations: *WHO* World Health Organization, *HIV* Human Immuno-deficiency Virus

Low-and middle-income countries (LMIC) are increasingly prioritising small and sick newborn care (SSNC) in national plans and targets [3], but the devices, and system changes required to implement high-quality inpatient newborn care are challenging [5, 7, 8]. In high-income settings, care of an admitted newborn often involves more than 20 devices [9]. Analyses of bottlenecks to scale-up of high-quality care for mothers and newborns in 12 LMICs with most of the world’s newborn deaths found that lack of devices and ward furniture was a major challenge, and healthcare workers were often not confident in their ability to use devices effectively [10].

Many hospitals, especially those in resource-limited settings, have a shortage of functional medical devices and supplies. Historically, donated medical equipment has been relied on to meet this need, often without considering local constraints for training for use, maintaining, repairing, and decommissioning equipment. This has resulted in “equipment graveyards”, [11, 12]. While donations can help address acute shortages, they often come without user training, sustained consumable supply, compatible spare parts, or maintenance plans to ensure continuous functionality [13–17]. Additionally, context-specific considerations affecting device performance (e.g., dust, extreme temperatures, humidity, frequent power outages, etc.) are crucial to ensure value for money based on lifetime use [12, 18]. To ensure continuous device functionality, planners need to include distribution, maintenance, and consumable costs. On-site warranties, although these may increase the purchase price, may provide better value long-term [10, 12, 19]. In addition to procurement, onsite training of both clinicians and hospital biomedical technicians is fundamental to effective and safe use of devices.

Costing of medical devices requires determining the device types and quantities needed and unit cost data [5, 12]. There is a lack of global normative guidance for device-to-baby ratios and furniture specifications needed to provide level-2 care [20]. We were unable to find any open-source costing tool to inform budgeting for neonatal care ward furniture and devices. Costing that considers other health systems inputs, such as infrastructure and human resources is also crucial, but requires wider systems costing, often with economic expertise [21].

### Aim and objectives

This paper is part of a supplement with the NEST360 Alliance, providing tools, analysis, and novel learning for implementing SSNC. In this paper, we aim to describe the development and refinement of a planning and costing tool. The intended audience is health planners at national, sub-national, or facility levels charged with planning and budgeting. The paper addresses three objectives:

- Objective 1: Review relevant planning and costing tools to assess content, design and costing approaches.
- Objective 2: Co-design content for a device and furniture planning and costing tool for a functional level-2 neonatal unit.
- Objective 3: Refine and use tool to estimate cost of national scale-up in mainland Tanzania.

## Methods

### Methods by objectives

#### Objective 1: Review relevant planning and costing tools to assess content, design and costing approaches

We searched for open-source planning and costing tools relevant to maternal and child health, including grey literature due to limited results in international bibliographies databases. Data extraction was informed by an adapted ISPOR Criteria for Cost(-Effectiveness) Review Outcomes (CiCERO) Checklist [22] (Additional file 1) and was completed in two phases. In phase 1, we compared data inputs, tool’s features, and economic approaches of widely used costing tools with maternal and child health fields to inputs required for care of small and sick newborns specifically. In phase 2, we examined the activity-based costing approach to establish its inclusion in the tool design, and device-specific planning and costing tool, UNICEF Oxygen System Planning Tool, to gain insight into its underlying costing assumptions, outputs, and format.

#### Objective 2: Co-design content for a device and furniture planning and costing tool for a functional level-2 neonatal unit

We engaged a multidisciplinary group of newborn care implementers with professional experience in LMIC (including clinicians, biomedical technicians and engineers, national policymakers, trainers, economists, and researchers) to inform tool development and ensure a user-friendly design. We defined criteria for the tool a priori and designed the branching logic framework to guide content and data inputs.

**Fig. 1:**
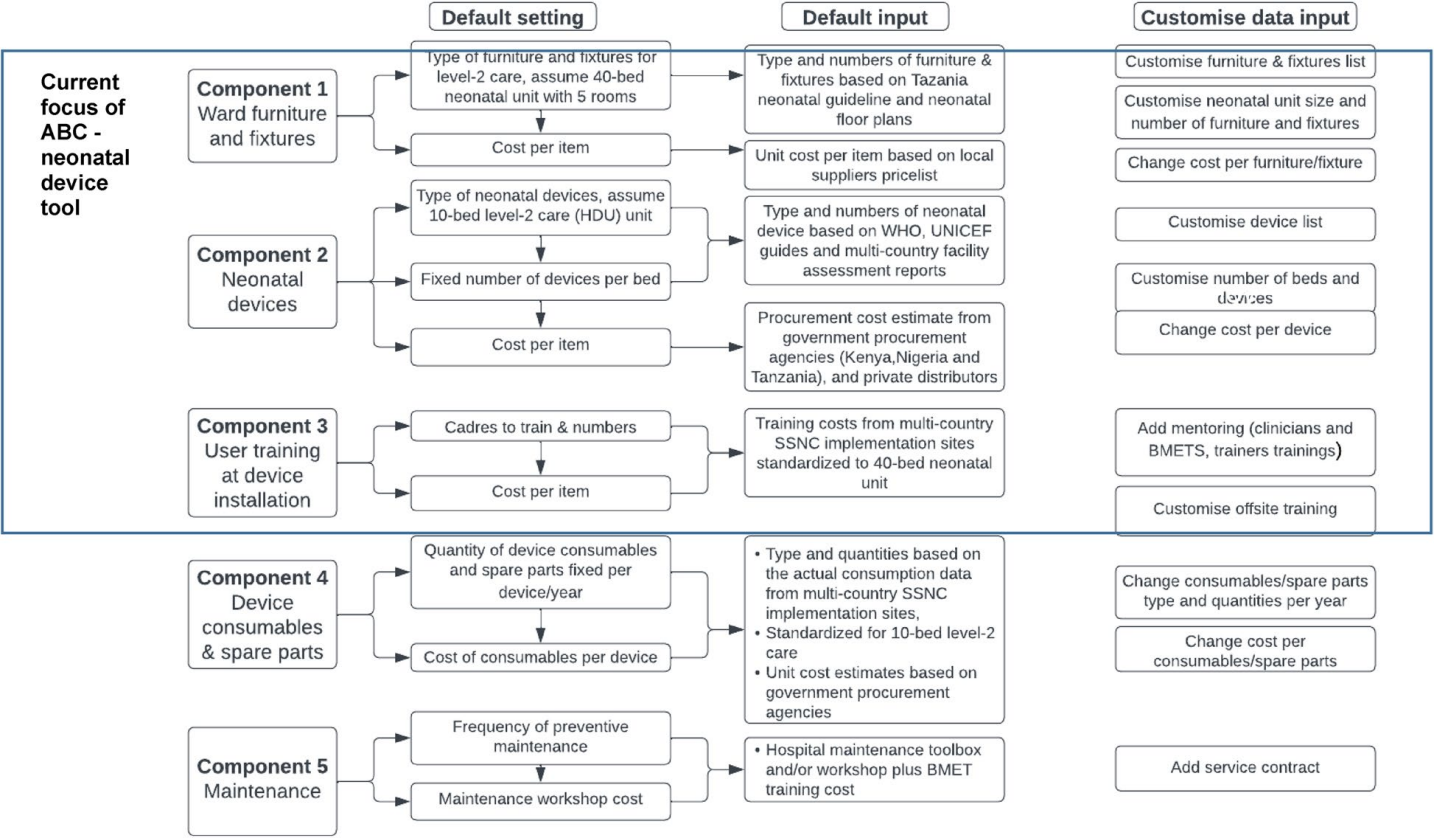
Activity-based costing (ABC) neonatal device tool’s overall structure with current focus on set up components (Components 1–3) Abbreviations: HDU; High Dependency Unit, BMET; Biomedical Equipment Technician, SSNC; small and sick newborn care

A priori criteria included:

- A default 40-bed neonatal unit, including a ten-bed level-2 unit for care of small and sick newborns, was adopted from the Tanzanian newborn unit floor plan for a District hospital. In Tanzania, this level-2 care unit is known as the high dependency unit (HDU). The floor plan includes five rooms: the HDU/ level-2 care (ten cots), a general, step-down neonatal ward (ten cots), an isolation room (five cots), a kangaroo mother care (KMC) room (ten adult beds) and a rooming-in area (five adult beds) [23] (Table 2). The default neonatal unit and level-2 care bed capacity can easily be adjusted up in multiples of ten.
- Remit to include the following components: (1) ward furniture and fixtures (permanently installed equipment or furniture in the newborn unit), (2) neonatal devices, and (3) training for use (Fig. 1). The tool was structured based on these three components and built in Microsoft Excel. Additional components were identified (i.e., (4) consumables and spare parts, and (5) maintenance) but not possible to include in this version of the tool given cost data gaps. Quantities required for the ward furniture and fixtures (first component) and user training at device installation (third component) were determined based on a default 40-bed neonatal unit. On the other hand, quantities required for the devices (second component) were based on the ten-bed level-2 care unit only (Table 2).

**Table 2:** Quantification and costing components incorporated in the tool for a default 40-bed neonatal unit, including a ten-bed level-2 (high dependency unit) for small and sick newborn care

		**Quantities and cost estimates for each of the following components included in the tool?**		
**Room**	**No. of beds**	**Furniture & Fixtures**	**Devices**	**User Training**
High Dependency Unit (Level-2)	10 cots	√	√	√
General Neonatal Ward (Step-Down)	10 cots	√	Basic only*	√
Isolation Room	5 cots	√	Basic only*	√
Kangaroo Mother Care Room	10 adult beds	√	Basic only*	√
Rooming-In Area	5 adult beds	√	Basic only*	√
**Total**	**40 beds**			

^*^Basic devices include thermometers, weighing scales, measuring tapes

#### Component 1: Ward furniture and fixtures (types, quantification, and costs)

A list of ward furniture and fixtures required for a newborn unit was compiled through a review of the World Health Organization (WHO) [24], the United nation international children’s emergency fund (UNICEF) guidelines [25] and Tanzanian national neonatal care manuals [26]. There were sparse data from these policy documents to inform the quantity of each item required, so we estimated quantities based on either number of beds or rooms as per the Tanzanian national floor plans for a District hospital newborn unit [23], supplemented by United Nations (UN) materials [25, 27, 28] and experts' opinion. To derive unit costs for furniture and fixtures we reviewed quotations (2022) from local government suppliers and private distributors (national or international distributor who supplies a range of neonatal items) tender documents from Tanzania, Kenya and Nigeria and calculated a median cost and range. Median costs from local government suppliers and private distributors were compared to assess variation. The local government suppliers’ median cost was used to derive the tool’s default point estimate.

#### Component 2: Neonatal devices (specification, quantification, and costs)

To identify types and quantities of devices to include in the tool for ten-bed level-2 care for small and sick newborns at District hospital, we reviewed several documentary sources [25, 27–30] and considered expert opinion. Device specifications, especially relevant for purchasers and procurement officials, were informed by UNICEF-NEST360 Target Product Profiles (TPP) [31]. A TPP is an outline of desired product characteristics aimed at addressing the needs of a particular disease(s)/medical condition [32]. The UNICEF-NEST360 TPP process involved a consensus-driven approach with 103 key stakeholders from 22 countries who voted on product characteristics for 16 types of technologies across six categories of care: hydration, nutrition, drug delivery, jaundice management, point of care diagnostics, infection prevention and control, respiratory support, and thermal management [31]. Of these 16 product categories, a minimum of two product options from different manufacturers of NEST360-qualified devices [33] were included in the tool to achieve brand neutrality.

To derive the price per device for a level-2 unit for the care of small and sick newborns, we followed a normative approach utilising market prices outlined by the government procurement agencies’ publicly available pricelists and requested quotes from private distributors in Kenya, Nigeria, and Tanzania. Median costs from government procurement agencies and private distributors were compared to assess variation. The government procurement agencies median cost was used as the tool’s default unit cost point estimate. Aside from government procurement agencies and private distributors, we identified the types of devices available from the UNICEF supply catalogue, though unit prices were not included due to variation in logistical costs e.g. freight across countries.

#### Component 3: User training at device installation

Training is crucial for safe use and troubleshooting of devices. Based on our experience of implementing a SSNC package through the NEST360 alliance in 67 newborn units across four countries, we costed a one-day, facility-based training combining local health providers (clinicians and nurses) and hospital biomedical engineers and technician(s). We reviewed user training logs to inform the number of participants and copies of necessary training materials. To derive unit costs, we reviewed current government reimbursement policies and prevailing market costs (i.e., cost of stationeries, meals, refreshments, etc.) across three countries (Kenya, Nigeria, and Tanzania). Malawi (the fourth implementing country with NEST360) was excluded as it had achieved a national scale-up to exceed ENAP target 4. The median value was calculated and used as the tool’s default estimate. Some sparse data inputs were refined through expert consultation.

#### Objective 3: Refine and use tool to estimate cost for national scale-up in mainland Tanzania

The tool was used in mainland Tanzania, one of the few countries that met the 2015 Millennium Development Goal target 4 of reducing under-5 mortality by two-thirds. However, from 2010 to 2015, the national neonatal mortality rate (NMR) remained almost unchanged [34]. This stagnation and in keeping with ENAP target 4, mainland Tanzania’s national health strategic plan and One Plan III prioritised the scale-up of level-2 care in 80% of District hospitals (146) and all (25) Regional Referral hospitals by 2025 [35].

To achieve this target, the Ministry of Health sought to develop a national investment case for scale-up to the 171 hospitals, incorporating infrastructure, human resources, and other system costs [21]. As part of the investment case development, this tool was customised to estimate furniture, fixture, and device costs (assuming all items would be purchased new) for both the District hospital and Regional Referral hospital scale-up. As per the guidelines Regional Referral hospital was defined to provide full level-2 care for sick babies and partial level-3 care for critically ill newborns awaiting referral to the intensive care unit (i.e., level-3 care) for further treatment and management.

Incremental costs for District and Regional Referral hospitals were estimated separately in the tool due to differences in unit size, types and quantities of devices needed. Incremental quantities were estimated based on Tanzania’s national neonatal floorplans, District hospital costing assumed a 40-bed size with a ten-bed level-2 unit for care of small and sick newborns (i.e., the tool’s default quantities), whilst Regional Referral hospital costing was customised from the default to an 80-bed neonatal unit with 20-bed level-2 care unit (i.e., tool’s default list of items with more quantities plus an estimate of devices for partial level-3 care) [23]. The additional neonatal devices and furniture and fixtures were multiplied by the unit costs derived from Tanzanian national medical agency pricelist (2022) [36] and local government suppliers respectively. The unit costs were converted to US dollars (2022 average exchange rate of 2324 TZS per USD) before being entered to the tool. The cost per capita at both levels of care was calculated using the mainland Tanzanian population (2022) of 60 million [37]; whilst cost per birth was estimated using annual total birth (2020) of two million [35].

Data collection and report generation were led by a purposively sampled team of ten Tanzanians, representatives from the Ministry of Health and the NEST360 country team, with economists' support. The Tanzanian team (consisting of six clinicians, three economists, and two qualitative researchers) were engaged through informal and formal interviews to understand their experiences on usability, time burden, and perceptions of the tool’s content.

## Results

Our findings and learnings are summarised according to the three objectives:

### Objective 1: Review relevant planning and costing tools to assess content, design and costing approaches

The widely used costing and impact estimation tools; the Lives Saved Tool (LiST) costing module [38] and One Health [39] were reviewed. Appraisal of Activity-Based costing approach [19, 40] was done to inform the tool’s design. We also identified a useful device-specific tool to learn from, the UNICEF Oxygen System Planning Tool [41, 42].

LiST estimates lives saved through reproductive, maternal, newborn, and child health (RMNCH) interventions, and has more approximate costs to inform return on investment (ROI) estimates [43, 44]. One Health is led by WHO and designed to inform health systems planning towards universal health coverage on a wide scale: national or sub-national levels [38, 39, 45] to generate estimates of health impact and cost, useful for developing broad investment cases. Both tools cover a broad remit of services and tend to be more focused on inputs for essential newborn care (level-1 care) than level-2 care. This is explained by the limited detail on neonatal care costs, for example, LiST costing module includes a bag and mask resuscitation and a few other items, mostly antibiotics. Costs in both tools are classified as set-up or recurrent, and calculated as either total or incremental, following an ingredients-based or bottom-up approach; where each resource required for an intervention is identified and valued using reliable cost estimates e.g., international price lists. Such an approach is suitable for costing interventions with defined guidelines but is less useful for interventions where various ‘ingredients’ lack normative quantity standards, as is the case for level-2 newborn care. Both LiST and One Health calculate the costs of the capital items of health systems components (e.g., infrastructure, furniture, devices and programmatic) as a percentage of total intervention costs, whereas we required a tool that identifies and quantifies key components e.g., type of ward furniture, fixtures, and neonatal devices for level-2 newborn unit and calculates their incremental costs thus adopting the activity-based costing approach for this tool. The activity-based costing approach is a principle that seeks to identify specific costs items and assign them to the health services delivered to better understand and manage total costs. Also, both One Health and LIST tools require skills and training for use whilst our remit was to develop a tool that can be used by a non-economic expert (Additional file 2).

The UNICEF Oxygen System Planning Tool was designed for a wider, non-specialist user group and focuses on incremental costs, primarily for set-up, and uses a stepwise approach, with easily customisable data input fields and a results dashboard [41, 42] (Additional file 2). This tool’s format was most applicable to our target audience and remit. However, the micro-costing approach of entering data in different sheets may be time-consuming and demanding, prolonging the planning process.

### Objective 2: Co-design content for a device and furniture planning and costing tool for a functional level-2 neonatal unit

The user-designed tool follows the Activity-Based Costing (ABC) to provide quantification and cost based on three components (Fig. 1): (1) ward furniture/fixtures (18 default but editable items); (2) neonatal medical devices (16 product categories with minimum specifications for use in LMIC contexts); (3) user training at device installation. Results are displayed in summary dashboards. The procurement report auto-populates the summary of quantities per item to be procured and outlines minimally acceptable product specifications, including examples of device names and models that met an accepted qualification process. The cost report provides a summary of scale-up costs and costs per component, which enables assessment of cost drivers. Impact of the investment on the budget is also calculated and expressed as cost per capita (total costs divided by the county’s population) and cost per birth (total costs divided by the country’s annual total birth).

#### Component 1: Ward furniture and fixtures (types, quantification, and costs)

A total of eighteen default furniture items were identified based on UN guidelines and national policies. The quantities required are shown in (Table 3). Recommended quantities included one extra item of each type of fixture as a contingency in case of breakdown.

**Table 3:** Ward furniture and fixtures required for a default 40-bed neonatal unit, showing median unit cost using data from Kenya, Nigeria, and Tanzania

Ward furniture & fixtures for neonatal unit	Estimated quantities for a 40-bed neonatal unit^a^	Local suppliers’ median unit cost^b^ (US$)	Local government suppliers’ unit cost range (US$)
Baby cots	25	194	187 – 219
Adult beds	5	547	280 – 777
Special beds-KMC	10	600	280 – 777
Special Chairs-KMC	5	82	65 – 337
Room thermometer	5	900	800 – 1200
Room heater	5	64	61 – 67
Wall clock with seconds' hand	5	13	12 – 26
Bedside lamp for procedures	6	284	111 – 331
Emergency trolley	6	816	757 – 1598
Medicine trolley	6	168	117 – 562
Ordinary (instrument) trolley	6	126	70 – 138
Refrigerator with freezer compartment	3	421	229 – 2160
Medicine cabinets	1	210	210 – 605
Water distiller 5L/Hr	1	421	233 – 821
Oxygen cylinder (back up with humidifier and flow splitter)	3	320	175 – 691
Oxygen cylinder (small for transport)	3	244	65 – 338
Measuring tape	6	2	1 – 4
Voltage stabilizer and UPS	3	1150	582 – 2339

^a^Quantities estimated for a default 40-bed neonatal unit, consisting of five rooms, including a level-2 neonatal unit for small and sick newborn care/HDU

^b^Median of prevailing market price from Kenya, Nigeria, and Tanzania (see Additional File 1 for further detail)

Abbreviations: US$; United States dollar, KMC; kangaroo mother care, UPS; uninterrupted power supply, L/Hr; litres per hour

**Fig. 2:**
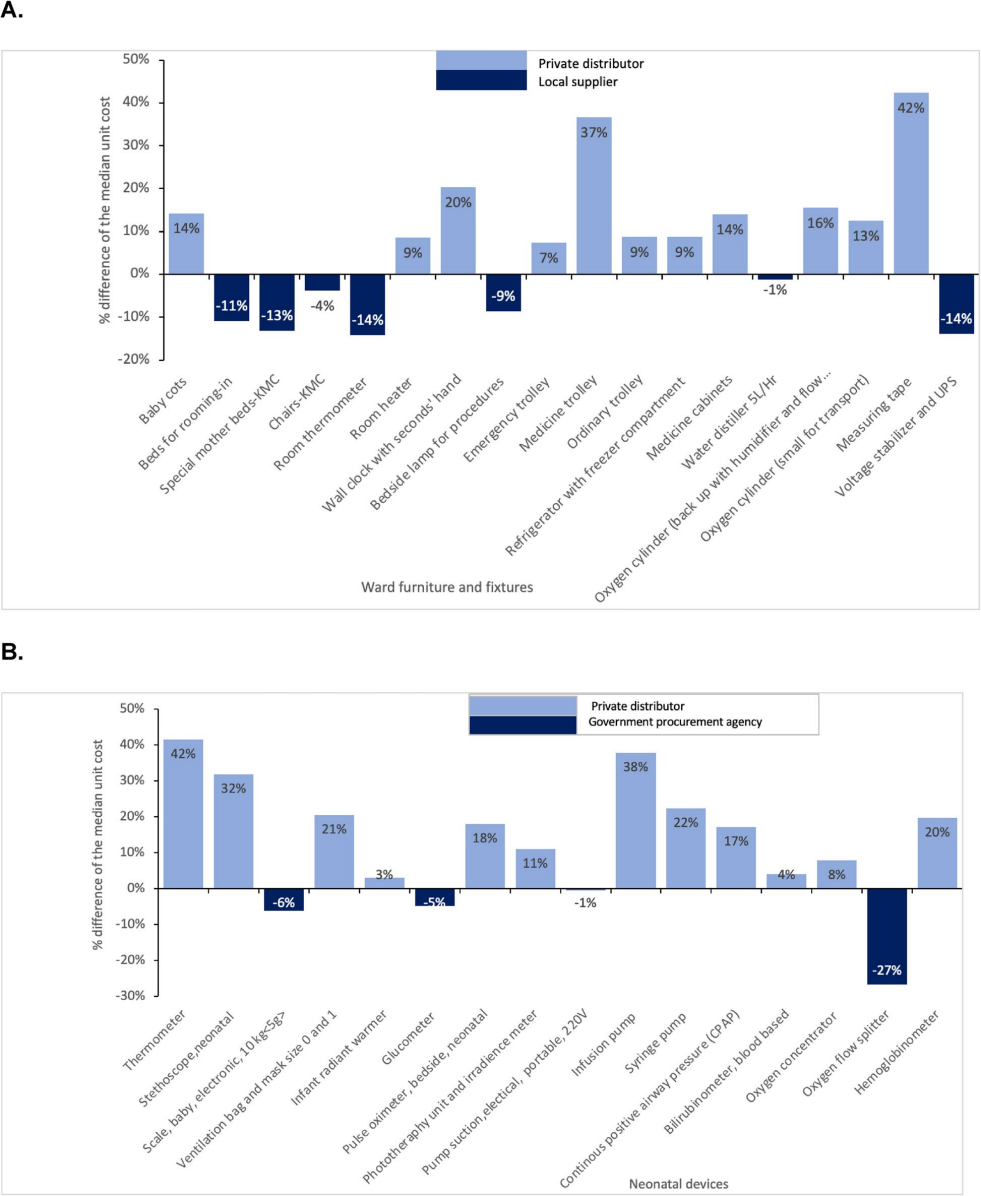
Relative percentage difference of median unit costs^a^ for furniture, fixtures, and devices required for a level-2 neonatal unit **A**. Compares local supplier median unit cost to private distributor median unit cost per ward furniture/fixture **B**. Compares government procurement agency median unit cost to private distributor median unit cost per device Footnotes: ^a^Median from Kenya, Tanzania, and Nigeria (see Additional File 1 for further detail) Abbreviations: UPS, uninterrupted power supply, KMC, kangaroo mother care, L/Hr

Unit costs were derived from local government suppliers' absolute price ranges across Kenya, Nigeria, and Tanzania. The costs were consistent for fifteen items except for voltage stabilisers and uninterrupted power supply (UPS) which were relatively higher in Kenya, and refrigerators with freezer compartments and emergency trolleys were expensive in Tanzania. Of the 18-ward furniture and fixtures items, 11 (61%) had greater median prices amongst private distributors compared to local government suppliers (Fig. 2a). The average relative percentage difference for the median cost of these 11 items was 17%, largely driven by the percentage difference in the cost of measuring tape between private distributors compared to local government suppliers (42%). On the other hand, seven (39%) items had lower median prices amongst private distributors compared to local government suppliers. The average relative percentage difference for the median cost of these seven items was 9% (Fig. 2a).

#### Component 2: Neonatal devices (device type and specification, quantification, and costs)

A default list of 16 types of devices was reached through review and consensus agreement among a multi-disciplinary group (Table 4). This default device list is editable to add or remove and was informed particularly by UN publications. Priority medical devices for newborn health from the WHO inter-agency list of (26) included forty-three devices according to the level of health care delivery, and use for laboratory, anthropometric, or general hospital context. UNICEF country-specific guides listed neonatal devices per level of newborn care: special care newborn unit (level-2 care neonatal unit) and essential newborn care unit (level-1 care newborn unit). The multi-country health facility assessment reports itemised devices available at District (level-2 care) and Tertiary hospitals (level-3 care) (see Additional file 3).

**Table 4:** Neonatal devices required for a default 10-bed level-2 unit for small and sick newborn care^a^, showing median device unit cost based on government procurement agencies in Kenya, Nigeria, and Tanzania

Medical devices for level-2 neonatal unit for small and sick newborn care	Estimated quantities for a 10-bed level-2 neonatal unit	Government procurement agencies’ median unit cost^b^ (US$)	Government procurement agencies' unit cost range (US$)
Stethoscope, neonatal*	5	29	21 – 67
Ventilation bag and mask size 0 and 1*	2	22	22 – 29
Infant radiant warmer*	2	3444	3333 – 3580
Glucometer	5	38	33 – 1342
Pulse oximeter, bedside, neonatal*	6	51	33 – 142
Phototherapy unit and irradiance meter*	2	1505	933 – 2167
Pump suction, electrical, portable, 220 V*	2	367	178 – 397
Infusion pump*	5	708	302 – 778
Syringe pump*	2	808	778 – 1375
Continuous positive airway pressure*	5	1333	1079 – 1833
Bilirubinometer, blood based	1	1944	1900 – 2000
Oxygen concentrator*	5	1000	933 – 1053
Oxygen flow splitter*	5	518	250 – 624
Hemoglobinometer	1	150	78 – 410
Thermometer^b*^	5	8	4 – 22
Scale, baby, electronic, 10 kg < 5 g > ^b*^	5	89	83 – 164
*Customized to add other devices as required*			

^a^Level-2 care is a high dependency unit in Tanzania

^b^Median from Kenya, Tanzania, and Nigeria (see Additional File 1 for further detail). Unit cost is inclusive of shipping cost, inspection cost, and distribution cost. Installation cost is excluded

^*^ Devices available on the UNICEF supply catalogue

Abbreviations: US$; United States dollar, HDU; high dependency unit

Device quantification assumptions were based on a ten-bed level-2 unit with an option to customise. UNICEF neonatal care manuals provided guidance on number of devices based on the number of beds: UNICEF India for a 12-bed unit (22); UNICEF Bangladesh for a ten-bed unit (24); and UNICEF Sierra Leone for a ten-bed unit (25). The total number of devices ranged between 21 and 30 and were classified as either essential or desirable (Additional file 3). Out of the sixteen, 11 of the device types, minimally acceptable specifications were included, based on UNICEF/NEST360 target product profiles [31], as well as two to three examples of device names and models that conformed to specifications [33] (Additional File 4).

Unit price was derived from government procurement agencies as the tool’s default estimate with an option to customise. Government procurement agencies' unit prices across the three countries were remarkably consistent for all the devices. The lowest to the highest unit price per device ratio averaged 0.28. However, the price difference of glucometers and syringe pumps was high in Tanzania, whilst phototherapy units and CPAP prices were high in Kenya. According to the government sources the costs included logistic costs (i.e., freight, inspection, duty, and distribution). Installation was assumed to be done by government-salaried biomedical experts.

Across the 16 devices, the private distributor median unit cost for 12 (75%) devices were higher than that of the government procurement agencies (Fig. 2b). The average relative percentage difference for the median cost of these 12 items was 20%. There was minimal difference (< 10%) in the median unit costs between government procurement agencies and private distributors for radiant warmers, bilirubinometers, and oxygen concentrators. The median unit prices for four neonatal devices (i.e., weighing scales, glucometers, suction pumps, and oxygen flow splitters) were found to be higher through government procurement agencies than with private distributors (Fig. 2b). In addition, thirteen of the sixteen devices were listed in the UNICEF supply catalogue. The glucometer, hemoglobinometer, and bilirubinometer instruments were not available.

#### Component 3: User training at device installation

We costed for training a minimum of 25 healthcare workers, including nurses, clinical officers, medical doctors, and biomedical engineers and technicians; neonatologists and neonatal nurse(s) could be included if available. The training was delivered by two external facilitators – a nurse and a biomedical engineer/technician, government-salaried employees. Costed items were facilitator fees and travel reimbursement, refreshments and meals, and training materials (e.g., job aids, flip charts, booklets, etc.) (Additional 4). Government per diem rates across the three countries were relatively consistent with ratio of lowest to highest per diem rate averaging 0.8. There was no variation in training input costs across countries. Data from 20 hospitals implementing with NEST360 showed nurses made up 50–75% of installation trainees drawn from newborn units, paediatric, maternity, and labour wards.

### Objective 3: Refine and use tool to estimate cost for national scale-up in mainland Tanzania

**Fig. 3:**
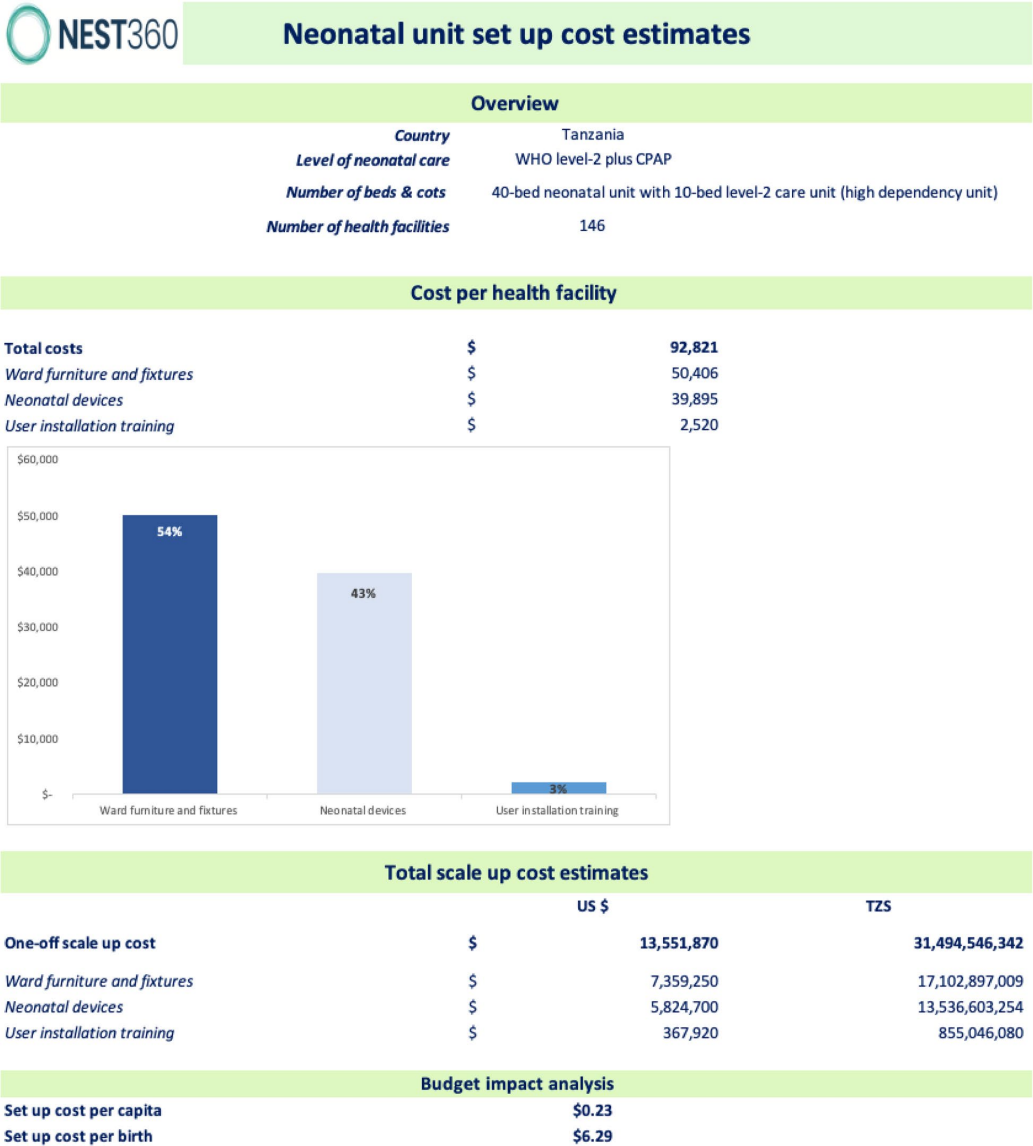
Illustrated tool output for scale-up of level-2 care in 146 District hospitals in mainland Tanzania. Abbreviations: HDU; High Dependency Unit, TZS; Tanzania shilling, US$; United States dollar, CPAP; continuous positive airway pressure

We estimated an incremental scale-up cost (assuming entirely new furniture and devices required for all categories) per District hospital at US$93,000 and US$346,000 per Regional Referral hospital. The higher cost of setting up a Regional Referral Hospital is attributed to increased unit capacity (10-bed to 20-bed level-2 care/HDU unit) that required more ward furniture, fixtures, and devices for level-2 care and additional devices for partial level-3 care. Partial level-3 care entails providing comprehensive inpatient special newborn care provided in secondary health facilities e.g., district hospitals plus some components of intensive newborn care services provided at the tertiary level of care, as defined by WHO (Table 1). Devices for partial level-3 care comprised a customised list of fourteen items (Table 5). The total cost to procure new furniture, fixtures, and devices and train users in the country's 171 hospitals was estimated to be US$22.2 million. Ward furniture and fixtures accounted for US$9.0 million (41%), neonatal devices for US$12.7 million (57%), and user training at device installation for US$0.5 million (2%). The incremental cost of the rollout to 146 District hospitals was US$13.6 million, with ward furniture and fixtures costing US$7.4 million (54%), neonatal devices costing US$5.8 million (43%), and user training costing US$0.3 million (3%) (Fig. 3). The total cost of scale-up for Regional Referral hospitals was slightly lower than District hospitals at US$8.6 million. District hospital national scale-up was estimated to cost an incremental US$0.23 per capita and US$6.30 per birth, while Regional Referral hospitals were estimated to cost US$0.14 per capita and US$4.10 per birth. The budget implications of scaling level-2 care to whole district coverage in the country estimated a 0.57% increase in government health expenditure per capita (from 40.62 in 2020). Adding 25 Regional Referral hospitals would be an additional 0.35%.

**Table 5:** Tanzanian customised neonatal devices for partial level-3 care of small and sick newborn care at Regional Referral hospital

Medical devices for level-2 neonatal unit for partial level-3 care for small and sick newborns^a^	Estimated quantities for a 20-bed level-2 neonatal unit^b^ for partial level-3 care	Government procurement agency unit cost^c^ (US$)
Incubator	2	7415
Ventilator (for neonates)	3	17,556
Laryngoscope set, newborn & 20 tubes	3	77
Apnea monitor	3	1287
Exchange transfusion kit	2	214
Retinotheraphy of prematurity screening device	1	343
Ultrasound for cranial & cardiac scans	1	7183
Total parenteral nutrition provision	1	214
X-ray machine	1	122,239
ECG unit, 3 channel, portable set	1	4218
Blood gas analyser	1	6197
Nebulizer machine	1	64
Examination Screen (X-ray box)	1	214
Neonatal transport system	1	1072

^a^Devices are specific for partial level-3 care only

^b^Quantities estimated are for a 20-bed level-2 neonatal unit (HDU) comprising level-2 care and partial level-3 care

^c^Unit costs were customised for Tanzania

Abbreviations: *ECG* Electrocardiography, *HDU* High dependency unit

Tool users in Tanzania had positive feedback on the stepwise tool structure and default entries of quantities and unit cost. They cited the need for additional guidance notes next to the data input fields to aid prompt and accurate data entry. On the other hand, non-clinical users reported difficulty understanding certain device descriptions and specifications and needed additional clarification and support. These concerns were refined in the tool and validated via a virtual walk-through session with the users.

It took one week to collect and enter information (e.g., unit costs and demographic data) and generate the cost and procurement reports. All the users felt the reports were simple to interpret and could be incorporated into their budget planning processes.

## Discussion

Implementing high-quality inpatient care for small and sick newborns requires sufficient, robust, context-appropriate devices and planning for user training and maintenance [5, 16]. We developed a novel newborn-specific planning and costing tool to support the national scale-up of SSNC and used the tool in Tanzania. The tool uses the ABC approach yet mirrors the stepwise approach of the UNICEF Oxygen System Planning Tool [16]. The tool's default costing estimates are based on publicly available government procurement agency pricelists, which are marginally lower on average than private distributor costs where available but may not be of comparable specification or post-distribution support.

Health system costing tools (i.e., the WHO One Health tool and the LiST costing module) had limited newborn-specific device inputs [39], which is unsurprising given the relatively recent global focus on SSNC. However, if countries are to achieve international standards for quality of care for small and sick newborns in health facilities [24], planners need to be enabled to more accurately budget and develop investment cases to mobilise resources. SSNC investment cases that used the LiST costing methodology had to add many specific items and cost assumptions to account for programme management and health system costs [43]. Our planning and costing tool is based on the activity-based costing approach, providing specific inputs for SSNC components, addressing a gap in existing tools. Incorporating newborn cost input data from this tool into existing health system tools (which may include estimates for components not included in our tool (e.g., human resources) could make costing estimates more comprehensive. On the other hand, the tool can also be used by a non-economic expert for standalone planning to refurbish an existing neonatal unit or estimate costs to fully fit out a new unit.

Use of the tool in mainland Tanzania revealed feasibility for use by a non-economic expert to generate reports and results were able to catalyse additional investment in care for small and sick newborns. To scale up SSNC nationally in Tanzania, the estimated incremental cost per capita was US$0.23 for 146 District hospitals and US$0.14 for 25 Regional Referral hospitals. These are modest estimates when compared to scaling up community-based maternal and newborn care interventions in Tanzania (US$1.30), Ghana (US$0.40), and Malawi (US$1.00) [46]. Similarly, when compared estimates of costs per capita to scale-up of mental health in low-income countries (US$1.85–2.60) [47] and malaria (US$1.20–5.70) services [48], the indicative SSNC estimates are relatively lower, suggesting it may be an affordable investment. Advocating and prioritising for sustained investment in SSNC requires tools that provide evidence-based cost estimates and impact on budget. However, many countries still rely on short-term donor investment for maternal and child health interventions, making planning for activities that span over several years challenging [49, 50], and newborns have been historically under-represented in health budgeting decisions [51]. Despite the disproportionately high neonatal mortality burden, and the potential return on investment in SSNC (between US$8 –12 for every US$1 invested) [21], the percentage of RMNCH donor aid that specifically mentions newborns remains small [52]. Costing estimates from the tool may help to catalyse more funding, from government sources and non-traditional donors.

Strengths of this work include the user-centric approach, systematic development of the tool, and transparency of the data sources. The tool’s customisability to reflect the local guidelines and costs, given the observed price variability and step-by-step approach with guidance notes, makes it feasible for use by individuals without an economics background. Reports generated by the tool, include minimum technical specifications of devices with corresponding product examples which can be useful to inform tender documents and budget cycle discussions. Procuring context-appropriate devices (i.e., those qualified for low-resourced settings) can increase the lifetime of a device and avoid exacerbating “equipment graveyards” [33]. These reports are possible to generate within a week, as found in Tanzania.

There are also limitations, inherent to data gaps for evidence-based newborn-to-device ratios. These gaps reflect the recent rise of SSNC on the global agenda – the first WHO norms and standards were only published in 2020 [24]. Whilst we reviewed existing information, device ratios in the past have tended to rely on expert opinion – highlighting crucial implementation research questions. The current version of the tool is focused on the set-up costs for a level-2 neonatal unit with device quantities based on a default ten-bed capacity. While the ten-bed option is pragmatic, most district hospitals will likely require more data on the need versus population or case mix to inform their decisions on the cots and bed capacity and device ratios. Important cost components (Fig. 1) such as device consumables, spare parts, and maintenance are not included yet and require more data. Ongoing data collection in hospitals implementing with NEST360, and in other regions and countries, may provide better quality data to assess device and staffing ratios and optimal unit size, including appropriate number of cots and beds. While we recognize that the current Excel version is user-centred, it may crash as more data is added; moving to a user-friendly platform may increase use.

We assumed a constant unit cost, whilst economies of scale and other cost-saving opportunities might exist, so the costs may be lower than we report. The tool default cost estimates may underestimate costs as the government procurement agencies, whilst mostly consistent across these three African countries, were generally lower than private distributors. Cost differences may be attributable to more stringent device specifications and explicit add-ons (e.g., installation, one-year warranty costs, starter packs of consumables, etc.) included in private distributor costs. The government procurement agency pricelists do not give minimum specifications for devices. Instead, some provide a list of companies from which health facilities can procure devices [53]. Research comparing actual costs incurred and default estimates beyond the three countries will improve the reliability and transferability of default estimates.

## Conclusion

Use of data to guide budget allocation and catalyse resource mobilisation is crucial in all contexts, but even more so in high-burden settings with relatively low per capita expenditures on health. The review revealed gaps for systematic planning tools notably for devices for small and sick newborn care. Our new tool provides practical evidence-based guidance to countries scaling up care for small sick newborns to accelerate progress toward ENAP and the SDG 3.2 target for neonatal survival. Systematic planning and costing could prevent costly mistakes that can arise from incorrect estimations (e.g., not enough devices procured, or devices not adequately maintained). To provide high-quality newborn care, the use of appropriate devices, furniture, and fixtures must be coupled with effective implementation including infrastructure, health workforce and data to drive change, reaching every district in every country.

## Supplementary Information

**Additional file 1. **Adapted ISPOR Criteria for Cost(-Effectiveness) Review Outcome (CiCERO) checklist.

**Additional file 2. **Review on planning and costing tools.

**Additional file 3. **Review on type and quantity of neonatal devices.

**Additional file 4. **ABC planning and costing tool for ward furniture and neonatal devices.

## Data Availability

Data used was available in the public domain. The Excel-based tool is available on www.newborntooolkit.org.

## Abbreviations

ABC: Activity-Based Costing
CiCERO: Criteria for Cost(-Effectiveness) Review Outcomes
ENAP: Every Newborn Action
HDU: High Dependency Unit
ISPOR: The Professional Society for Health Economics and Outcomes Research
KMC: Kangaroo Mother Care
LiST: Lives Saved Tool
LMIC: Low- and Middle-Income Countries
NEST360: Newborn Essential Solutions and Technologies
RMNCH: Reproductive Maternal Newborn and Child Health
ROI: Return on Investment
SDG: Sustainable Development Goal
SSNC: Small and Sick Newborn Care
TPP: Target Product Profile
TZS: Tanzania Shilling
UN: United Nations
UNICEF: United Nations Children’s Fund
US: United States
WHO: World Health Organization
