# Supplementary material for: Devices and furniture for small and sick newborn care: systematic development of a planning and costing tool
Source: BMC Pediatr. 2023 Nov 15;23(Suppl 2):566. doi: 10.1186/s12887-023-04363-w (PMC10652422; doi:10.1186/s12887-023-04363-w)
Supplement: Supplementary file 1 — Additional file 1. Adapted ISPOR Criteria for Cost(-Effectiveness) Review Outcome (CiCERO) checklist. [file 12887_2023_4363_MOESM1_ESM.pdf]

ISPOR CiCERO Checklist:
Criteria for Cost (-Effectiveness) Review Outcomes For systematic literature reviews that summarize cost and cost-effectiveness outcomes

The ISPOR CiCERO Checklist is a tool to assess the quality and risk of bias in systematic reviews of cost and cost-effectiveness outcomes. Stage six was the only applicable step for the review of the planning and costing tools.

Adapted criteria used to review the tools:

1. Focus on maternal and child health and devices.
2. Calculates cost of scale-up and unit cost e.g., per service and total cost by the level of care.
3. Study perspective
4. Scope of costs; Includes the cost of capital investments.
5. Has default estimates.
6. Country where the tool has been used.
7. Availability of user guide.
8. Publicly available software.

Stage 6. Presentation and reporting	Possible answers
**Question 11. Were the original studies included in the review described in adequate detail?**	*Y*
Comment:<br>*Answer "NA" for each sub-question of question 11 if no studies were identified.*	
**The reviews should report the following points for each of the included studies:**	
**11.1. Country of studied population**	*Y*
**11.2. Description of the population of analysis**	*Y*
**11.3. Time horizon, study perspective**	*Y*
**11.4. Discount rate**	*Y*
Comment:<br>*Answer NA if only short-term trials were involved, ie, one-year horizon or less.*	
**11.5. Adjustment of inflation**	*Y*
**11.6. Interventions compared**	*NA*
Comment:<br>*Answer "NA" if comparing interventions was not an objective of the review (eg, cost-of-illness / burden of disease)*	
**11.7. Method(s) for valuation of economic outcomes**	
**(a) Cost(s) in the healthcare sector according to the horizon of interest (direct costs, capital costs)**	*Y*
**(b) Indirect medical costs**	*NA*
**(c) Costs outside the healthcare sector, such as productivity loss (indirect costs)**	*NA*
**11.8. Method(s) for valuation of effectiveness outcomes, including source, type of source, estimates, duration (when relevant)**	*Y*
Comment:<br>*Answer "NA" if assessing cost-effectiveness was not an objective of the review (eg cost-minimization, cost-of-illness / burden of disease or other costs analysis).*	
**11.9. Compliance/adherence with treatment**	*NA*
Comment:<br>*Answer "NA" if the review has a top-down macro-level approach or if the review analyzes an intervention performed without any follow up.*	
**11.10. Decision analytic modelling or approach to calculation of economic outcomes**	*NA*
Comment:<br>*Answer "NA" if the review includes only within-trial cost or cost-effectiveness studies.*	

**11.11. Cost outcomes and/or health outcomes, eg, gained life years, number of deaths avoided, or QALY, and outcomes of economic value of an intervention, eg ICER or INHB.**	*N*
**11.12. Uncertainty**	*Y*
Comment: *Answer "Yes" if the review reported whether analyses are deterministic or probabilistic or based on other types of simulation.*	
**11.13. Conflicts of interest and sources of funding**	*NA*
**11.14. Software used (R, STATA, SAS, Excel, SPSS etc)**	*Y*
