# Supplementary material for: Devices and furniture for small and sick newborn care: systematic development of a planning and costing tool
Source: BMC Pediatr. 2023 Nov 15;23(Suppl 2):566. doi: 10.1186/s12887-023-04363-w (PMC10652422; doi:10.1186/s12887-023-04363-w)
Supplement: Supplementary file 2 — Additional file 2. Review on planning and costing tools. [file 12887_2023_4363_MOESM2_ESM.pdf]

Review of costing tools

A. Tool's review

	One health tool (OHT)[1]	Lives saved tool (LiST) costing module[2, 3]	UNICEF Oxygen System Planning tool (OSPT)[4, 5]
**Description of the tool**	Provides a single mechanism for integrated planning, costing, and budgeting of health sector priorities, including health system strengthening strategies to inform national strategic health planning.	LiST is a deterministic mathematical model that estimates both the financial cost of scaling up and the resulting impact on stillbirths and maternal, newborn and child deaths.	The tool estimates oxygen demand and quantifies equipment and supplies for health facility
**Purpose**	Facilitates assessment of wide- sector costs related to the areas of maternal, newborn, and reproductive health, child health, vaccination, malaria, tuberculosis, HIV/AIDS, nutrition, and water sanitation and hygiene,in addition, it contains modules for human resources, infrastructure, logistics, financial space, programme and channel analysis, intervention coverage and costing, bottleneck analysis, and programme costing,	Promote evidence-based resource allocation decisions and aid in planning for the expansion of maternal, neonatal and child health interventions at policy and strategic planning levels.	Allows planning of oxygen supply system from the oxygen source to the patient delivery device at different levels and placement of new oxygen plants.
**Users**	National health planners	National, international health planners	Planners at the national, subnational or health facility level
**Tool's input**	Epidemiological data, baseline and targeted intervention coverage, health program activity requirements, health system requirements and costs of commodities and program costs	Target population, population in need, coverage, and delivery channels	Inputs required are:<br>○ Health facility data<br>○ Oxygen plant data<br>○ Country inputs
**Software**	The tool is part of the Spectrum suite of software models, and overseen by the UN InterAgency Working Group on Costing (IAWG-Costing) led by WHO.	Part of the Spectrum software, maintained by Avenir health. Data can be copied to excel for further manipulation.	Built-in Excel with tabs on: tool's introduction, instructions, resources, country inputs, health facility dataset, oxygen plan dataset, output dashboards, source recommendations

**Methodology**	Bottom up, ingredient-based approach.<br>Allows for estimating historical or normative costs of providing health services.<br>Default but editable estimates of the treatment assumptions for each intervention – more than 400 interventions, grouped into 12 health programs: maternal/newborn and reproductive health, child health, immunization, malaria, tuberculosis (TB), HIV/AIDS, nutrition, Water, Sanitation and Hygiene (WASH), non-communicable diseases (NCDs), mental neurological and substance abuse disorders, adolescent health, and neglected tropical diseases	The module uses an ingredients approach based on four components: personnel and labour; drugs and supplies; other recurrent costs; and capital costs.<br>Infrastructure investment allocated proportion for projection.<br>Allows manipulation of currency and inflation rate<br><br>LiST interacts with other modules, the AIDS Impact Module, Family Planning Module and Demography Projections Module (Dem Proj),	Overhead costs (shipping, customs, distribution, installation, maintenance, and training) are calculated as a % of capital equipment costs and can easily be set by the user.<br>Oxygen demand quantification based on the number of beds.<br><br>Allows for the planning of multiple facilities (1-1000).<br>Permits scenario modelling.<br>Costings given in $ USD.
**Default estimates derivation**	Global/regional defaults for all values derived from Standard WHO protocols and expert opinion to specify the type and quantity of inputs.<br>Costs derived from WHO CHOICE, international drug prices (UNICEF supply catalogue and MSH international drug price indicator)	Default based on other tools: One Health, WHO CHOICE (staff baseline data on salaries, benefits, and time utilization), and WHO norm and guidelines where available.	Default cost estimates based on UNICEF Supply Catalogue with option to edit especially if not procuring through UNICEF
**User support**	Publicly available detailed manuals, help screens, and tutorials to guide the use of the tool.	Detailed manuals and tutorials to guide the use of the tool.	Guidance notes on how to fill the tool (sets & defines input parameters)<br>Colour-coded fields showing essential, desired, and optional fields for data inputs
**Tool results**	The tool provides planners with a single framework for estimating health impact, health system investments and service outputs, total and incremental costs by program area and resource type for baseline and projection years, program costs, bottleneck analysis, budget mapping, financial space and financial projections.	Used in conjunction with LiST module to Scenarios to estimate costs, coverage rates of interventions and program costs.	Total cost of ownership, operational costs,<br><br>Oxygen equipment list, consumables, and diagnostics devices

**Assumptions**	Safety stock (as percentage of need), allowing set of targets for how much gap will be filled.	Salaries are assumed to be annual salary for a full-time staff person.<br>Benefits are calculated as a percentage of those salary costs.<br>Changes in distal variables such as increases in per capita income or higher levels of maternal education will affect mortality by increasing coverage of interventions or reducing risk factors.	Considers unforeseen situation e.g., pandemics and develop scenarios (shipping costs increased by 30%)
**Limitations**	OHT is not open-source- calculations,formulas, and assumptions are not open to users thus cannot be easily traced or changed by a user.<br>Not suitable for a granular, detailed costing analysis at the facility level.<br>Although manuals and documentation exist and OHT training have been conducted in many LMICs, the tool software is less approachable for Ministry of Health employees with limited capacity and more familiarity with programs like Excel.<br><br>Medical devices are per level of care and the unit cost estimates are limited	Default estimates are provided at a global level, with no variation for different countries.<br><br>Data availability- if no baseline, can't evaluate impact accurately.<br>User friendliness<br>Sensible scale up targets	The micro-costing approach of entering data in different sheets may be time-consuming and demanding, prolonging the planning process.

B. Features of the Oxygen planning tool

- An Excel-based tool used at different administrative levels.
- Estimate capital and operating costs for oxygen therapy products.
- Allows the development of multiple scenarios.
- Provides guidelines to help define the input parameters.
- Different planning option tabs
- Geo-location features
- Clearly categorise data inputs

- In-built default estimates, customizable to reflect local context
- Output tab to visualize results.

References

1. Avenir Health. *OneHealth Tool*. [cited 2022 5/5/2022]; Available from: https://www.avenirhealth.org/software-onehealth.php.
2. Bollinger, L.A., et al., *Lives Saved Tool (LiST) costing: a module to examine costs and prioritize interventions.* BMC Public Health, 2017. **17**(S4).
3. Walker, N., Y. Tam, and I.K. Friberg, *Overview of the Lives Saved Tool (LiST).* BMC Public Health, 2013. **13**(Suppl 3): p. S1.
4. UNICEF. *Oxygen System Planning Tool: Quick Reference Guide and FAQ*. [cited 2022; Available from: https://www.unicef.org/innovation/media/13711/file/UNICEF%20Oxygen%20Planning%20Tool.pdf.
5. UNICEF. *Oxygen System Planning Tool*. 2020 [cited 2022; Available from: https://www.unicef.org/innovation/oxygen-system-planning-tool.
